# CX3CR1 Signaling on Monocytes Is Dispensable after Intracerebral Hemorrhage

**DOI:** 10.1371/journal.pone.0114472

**Published:** 2014-12-03

**Authors:** Roslyn A. Taylor, Matthew D. Hammond, Youxi Ai, Lauren H. Sansing

**Affiliations:** 1 Department of Immunology, University of Connecticut Health Center, Farmington, Connecticut, United States of America; 2 Department of Neurology, Yale University School of Medicine, New Haven, Connecticut, United States of America; 3 Department of Neuroscience, University of Connecticut Health Center, Farmington, Connecticut, United States of America; University of Michigan, United States of America

## Abstract

Intracerebral hemorrhage is a subset of stroke for which there is no specific treatment. The Ly6C^hi^ CCR2^+^ monocytes have been shown to contribute to acute injury after intracerebral hemorrhage. The other murine monocyte subset expresses CX3CR1 and lower Ly6C levels, and contributes to repair in other disease models. We hypothesized that the Ly6C^lo^ CX3CR1^+^ monocytes would contribute to recovery after intracerebral hemorrhage. Intracerebral hemorrhage was modeled by blood injection in WT and CX3CR1-null bone marrow chimeras. Neurological outcomes and leukocyte recruitment were quantified at various time points. Functional outcomes were equal at 1, 3, 7, and 14 days after intracerebral hemorrhage in both genotypes. No differences were observed in leukocyte recruitment between genotypes on either 3 or 7 days after intracerebral hemorrhage. A few hundred Ly6C^lo^ monocytes were found in the ipsilateral hemisphere in each genotype and they did not change over time. Peripherally derived CX3CR1^+^ monocytes were observed in the perihematomal brain 7 and 14 days after intracerebral hemorrhage. Our data suggests CX3CR1 signaling on monocytes does not play an influential role in acute injury or functional recovery after intracerebral hemorrhage and therefore CX3CR1 is not a therapeutic target to improve outcome after intracerebral hemorrhage.

## Introduction

Intracerebral hemorrhage (ICH) is a devastating subset of stroke that has a 30–50% mortality rate within the first 30 days [1]. Currently, there is no specific treatment for ICH, therefore finding a therapeutic target to limit secondary injury is critical [2]. ICH often results from hypertension-induced rupture of weakened blood vessels within the brain [3]. The exposure of brain tissue to a mass of blood components causes an inflammatory response through microglial activation and the recruitment of peripheral blood leukocytes into the perihematomal region [3]. Blood-derived monocytes enter the ipsilateral hemisphere as early as 12 hours after ICH and constitute the largest population of peripheral leukocytes in the brain at 12 and 72 hours [4], [5]. There are two main subsets of monocytes in mice–the inflammatory monocytes, which express CD11b, high levels of Ly6C, and the chemokine receptor CCR2, and the patrolling monocytes that express CD11b, low levels of Ly6C, and the chemokine receptor CX3CR1. Our lab has recently shown that the Ly6C^hi^, CCR2^+^ monocytes contribute to early injury after ICH [5].

CX3CR1 is a chemokine receptor found on microglia and the Ly6C^lo^ monocyte subset [6]. At steady state, the Ly6C^lo^, CX3CR1^+^ monocytes crawl along and patrol the endothelium [7]. The Ly6C^lo^, CX3CR1^+^ monocytes are classically known as the “resident monocytes” and are associated with a healing phenotype [8], [9]. This subset of monocytes has been shown to play a pivotal role in recovery from spinal cord injury [10], [11], myocardial infarction [12], and excitotoxic brain injury [13]. However, conflicting reports suggest improved late recovery after spinal cord injury in chimeric mice with CX3CR1-deficient monocyte-derived macrophages [14].

The ligand for CX3CR1, CX3CL1, is constitutively expressed by neurons and soluble CX3CL1 is increased after brain injury [15]. In patients with acute ischemic stroke, higher plasma CX3CL1 is independently associated with better outcome [16]. In mouse models of cerebral ischemia, exogenous CX3CL1 reduces infarct size and improves long-term outcomes, [17], [18] although it is unclear whether these effects are mediated by microglia or blood-derived monocytes. In a model of kainic acid-induced excitotoxic brain injury, the Ly6C^lo^, CX3CR1^+^ monocytes migrate to the injured brain and reduce neurological disability and neuronal degeneration, suggesting these monocytes have a role in neuroprotection [13]. Currently, the role of the Ly6C^lo^, CX3CR1^+^ monocytes in injury and recovery after ICH is unknown. Based on these other models, we hypothesized that the Ly6C^lo^, CX3CR1^+^ monocytes would migrate into brain towards CX3CL1 and play an important role in functional recovery after ICH at sub-acute time points (days 3–14). Our results suggest that CX3CR1 on monocytes does not play an influential role in acute inflammation or functional recovery after ICH.

## Materials and Methods

### Animals

Male B6.SJL-Ptprca Pep3b/BoyJ (CD45.1), C57/BL6J (WT), and B6.129P–Cx3cr1tm1Litt/J (CX3CR1^GFP/GFP^) mice were purchased from The Jackson Laboratory and then were bred in house. All mice were housed in standard conditions with a 12 hour light/dark cycle (lights on from 7∶00 am to 7∶00 pm), 22.2°C, and *ad libitum* access to food and water. All mouse procedures were completed with approval of the University of Connecticut Health Center Animal Care and Use Committee and were in compliance with the National Institutes of Health (NIH) Guide for the Care and Use of Laboratory Animals (Protocol number: 2010-0658).

### Generation of bone marrow chimeras

Bone marrow (BM) chimeras were created in order to specifically study the role of CX3CR1 on monocytes [19]. Wild-type (WT) CD45.1 mice 5–6 weeks of age were irradiated with 1200 rads and reconstituted the same day with congenically marked CD45.2 bone marrow cells either from a WT or a CX3CR1^GFP/GFP^ mouse, herein referred to as WT BM chimeras and CX3CR1-null BM chimeras, respectively. The use of CD45.1 and CD45.2 WT cells allows for the differentiation of resident cells of the nervous system from blood-derived leukocytes. BM cells were allowed to engraft for 8–9 weeks prior to ICH.

### ICH Surgery

Mice were anesthetized and maintained with 1–5% isoflurane and all efforts were made to minimize pain and suffering. ICH was modeled by injecting 20 µl of whole blood from a WT donor into the right striatum 2.5 mm lateral to bregma, 3 mm deep, at a 5° angle as previously described [20]. It has been previously shown that the cellular components within the blood used to create the ICH can impact the functional outcome and inflammatory response in the mice [21], therefore WT blood was used to create ICH for all experiments. WT and CX3CR1-null mice were 8–10 weeks of age when ICH was induced. For the chimera experiments, the mice were irradiated at 5–6 weeks and then cells were allowed to engraft 8–10 weeks prior to ICH, therefore mice were 13–15 weeks of age when ICH was induced.

### Behavior Tests

Mice were behaviorally tested for 14 days after ICH to examine neurological recovery as previously described [20]. All behavioral testing was analyzed by an observer blinded to genotype. For cylinder test, mice were place in an open jar and allowed to freely rear for 20 rears on days 1, 3, 7, 10 and 14. Initial paw placement was recorded to calculate a laterality index (right−left)/(right+left+both) in which positive numbers indicate left sided weakness. For open field, mice were placed into the 16″×16″ field with an infrared beam grid and allowed to explore the chamber for 20 minutes; total number of beam breaks quantified total locomotor activity [20]. For forced run, mice were placed into an enclosed treadmill and forced to run at various speeds; the top speed that a mouse could run was recorded. Open field and forced run testing were performed once on day 7 or 14 to avoid learning effects. To test whether WT and CX3CR1-null mice have baseline differences in behavior, non-chimeric WT and CX3CR1-null mice were also test on cylinder test, open field, and forced run test.

### Flow Cytometry

Mice were sacrificed 3, 7 and 14 days after ICH. Brains were harvested for flow cytometry as previously described [20]. Brains were harvested, cerebellums removed, and hemispheres were separated, then mechanically and enzymatically digested. Leukocytes were isolated from the interphase of a 70%/30% percoll gradient. Cells were stained with a 1∶100 dilution of monoclonal antibodies specific for cell surface markers (eBioscience: CD45.1 (A20), CD45.2 (104), Ly6C (HK1.4); BD Biosciences: CD11b (M1/70), Ly6G (1A8), and CD3 (145-2C11)) prior to being run on a LSRII flow cytometer. Alexa Fluor 350 carboxylic acid succinimidyl ester (Invitrogen) was used to identify live and dead cells. Populations were gated on live/dead, singlets, forward and side scatter and then as follows: CD45.2^+^, Ly6C^hi^, Ly6G^−^, CD11b^+^ (inflammatory monocytes), CD45.2^+^, Ly6C^lo^, CD11b^+^ (resident monocytes), CD45.2^+^, CD3^+^ (T cells), CD45.2^+^, Ly6G^+^, Ly6C^+^ (neutrophils), CD45.1^+^, CD11b^+^ (microglia). For samples used for intracellular cytokine stain, cells were stained with surface markers as described above and then fixed and permeabilized using a commercially available kit (CytoFix/CytoPerm, BD Biosciences) for 20 minutes at 4°C. Cells were then stained with monoclonal anti-TNF-α (MP6-XT22, BioLegend) for 30 minutes at 4°C. Counting beads were added to samples prior to running and the sample recorded until event numbers dropped to few events/second. Cell population numbers were calculated by adjusting for counting beads recorded. To determine chimerism, flow cytometry was performed on blood samples. Blood was collected into tubes with 200U/mL heparinized-PBS and lysed twice prior to staining with cell surface markers described above. Percent chimerism was calculated by: Percent CD45.2^+^ cells/(percent CD45.2^+^ + percent CD45.1^+^ cells) (Figure S1).

### Immunohistochemistry

Mice were intracardially perfused with 150 µg of Texas Red-tomato lectin (Vector Labs) at a concentration of 1 mg/ml [22] at sacrifice 7 and 14 days after ICH. Mice were then perfused with 20 mL PBS followed by 20 mL 4% paraformaldehyde. Brains were removed and postfixed in 4% paraformaldehyde for 4 hours. Brains were submersed in 30% sucrose for 48 hours prior to being embedded in OCT and cut into 8 µm sections. Sections were blocked for 1 hour in 2% goat serum and stained for Ly6B.2-biotin (clone 7/4 1∶100; AbD Serotec) with a secondary streptavidin in Alexa Fluor 647 (1∶1000; Life Technologies) and Dapi. To control for unspecific staining Ly6B.2 staining, sections were stained with only secondary antibodies. Photographs were taken on an Axiovert 200M microscope (Zeiss) using the Axiovision LE program provided by Zeiss. Adjustments were made using Zen lite software.

### ELISA Assay

Mice were sacrificed 7 days after ICH and brains were harvested, hemispheres were separated and were immediately flash frozen. Hemispheres were homogenized with 1 mm glass beads and RIPA buffer (Cell Signaling) containing phosphatase inhibitors (Roche) in a bead beater. Samples were then spun at 8,000 rcf for 30 seconds followed by sonication and an additional spin at 14,000 rcf for 15 minutes. BCA assay was performed to quantify protein concentration and 80 µg of total protein taken from brain tissue was used per well for the IL-6 and TNF ELISA assays (eBioscience).

### Statistical Analysis

Based on means and standard errors of cylinder testing from prior work, an n = 7 is required to achieve 80% power to detect a 25% difference in laterality between groups at α = 0.05 [23]. Cylinder testing was evaluated by repeated measures ANOVA. Open field was evaluated by student’s t test. Preliminary results depicted top running speed is not normally distributed, therefore, top running speed was evaluated by Mann-Whitney U test. Analysis of leukocyte recruitment was analyzed by student’s t test.

## Results

### WT mice recover quickly from autologous blood injection

**Figure 1:**
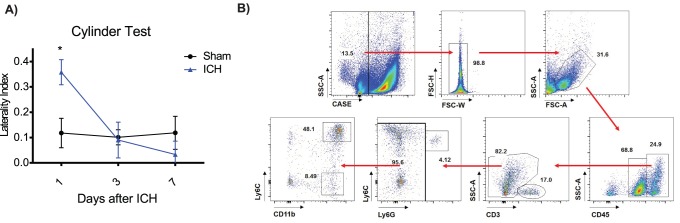
Ly6C^lo^ monocytes are present in the ipsilateral hemisphere 72 hours after ICH. **A)** WT mice were cylinder tested 1, 3, and 7 days after either sham or ICH surgery. Means graphed with s.e.m., n = 6–8, *p<0.05 **B)** Ly6C^lo^ monocytes are found in the ipsilateral hemisphere 3 days after ICH when mice recover by cylinder test. Cells were gated on live/dead, singlets, leukocytes were found by forward and side scatter, and then further gated to isolate populations as shown in these representative plots.

WT mice subjected to autologous blood injection showed a right forelimb preference twenty-four hours after ICH, indicating a left hemiparesis. By 72 hours, ICH mice have recovered equally to mice given sham surgery (Figure 1). Flow cytometry performed 72 hours after ICH to quantify the cellular infiltrate demonstrated 150.1±88.1 Ly6C^lo^ monocytes in the ipsilateral hemisphere (n = 6–8). We have previously reported that the Ly6C^hi^, CCR2^+^ monocytes contribute to early injury after ICH [5]. Based on the reported neuroprotective role of the Ly6C^lo^, CX3CR1^+^ monocytes in other injury models, we sought to determine whether these cells were contributing to recovery.

### Monocytes enter the ipsilateral hemisphere in a CX3CR1-independent manner

Since CX3CR1 deficiency on microglia has been implicated in enhanced neuroinflammation [6], we generated bone marrow chimeras to study the role of CX3CR1 specifically on peripheral leukocytes. We irradiated congenically marked CD45.1 mice and reconstituted them with BM from either WT or CX3CR1-null mice, which are both congenically marked by CD45.2, allowing the differentiation between host and donor cells. At 8 weeks, WT BM chimeras had 95.2±1% chimerism and CX3CR1-null BM chimeras had 94.03±0.9% chimerism, indicating that both genotypes engrafted the donor bone marrow successfully (n = 7–9). We then subjected the mice to ICH surgery to determine the role of Ly6C^lo^, CX3CR1^+^ monocytes over time. Based on the reports that the Ly6C^lo^, CX3CR1^+^ monocytes aid in repair after spinal cord injury and myocardial infarction after 7 days, we expected the monocytes to increase in numbers in the perihematomal brain during the second week after ICH. Immunohistochemistry was performed 3, 7 and 14 days after ICH, time points when the CX3CR1^+^, Ly6C^lo^ monocytes may be playing a role in tissue repair, to identify the location of the CX3CR1^+^ cells in the brain.

**Figure 2:**
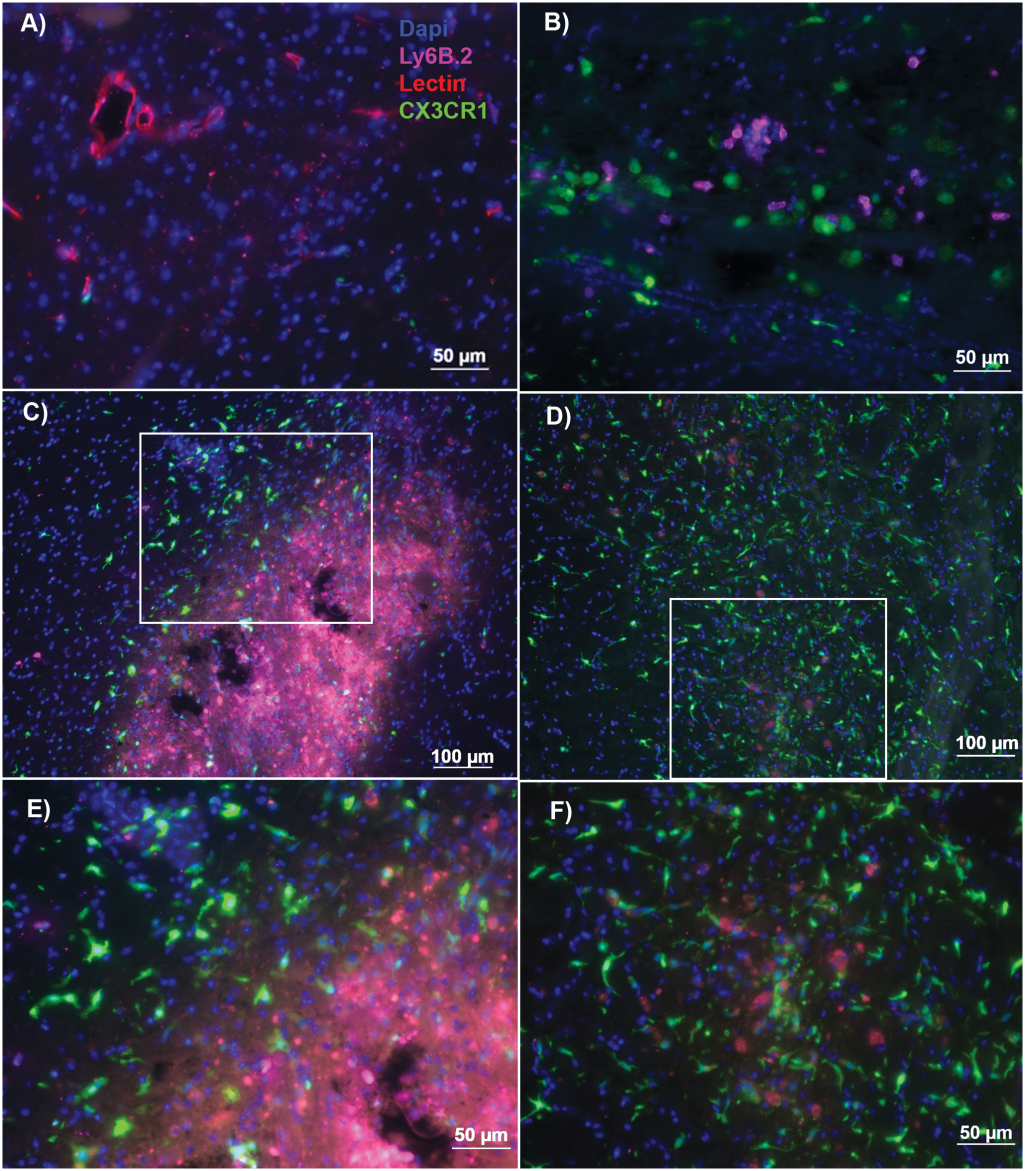
CX3CR1^+^, Ly6C^lo^ monocytes are present in perihematomal brain in CX3CR1-null BM chimeras. Mice were injected intracardially with 150 ug tomato lectin to delineate vasculature. Brain slices were stained with Ly6B.2 antibody to differentiate Ly6C^hi^ monocytes and neutrophils from Ly6C^lo^ CX3CR1^+^ monocytes and Dapi nuclear stain. **A)** Intracardiac injection of lectin stains vasculature in the brain– 20x. **B)** At 72 hours after ICH, distinct subsets of amoeboid Ly6B.2^+^ (Ly6C^hi^ monocytes or neutrophils) and GFP^+^ (CX3CR1^+^) monocytes are seen in the perihematomal region. 20x. **C)** At 7 days after ICH, amoeboid, Ly6B.2^−^,CX3CR1^+^ cells are seen in the brain parenchyma surrounding the ICH cavity. The center of the ICH cavity is filled with Ly6B.2^+^ (Ly6C^hi^) monocytes or neutrophils. Tomato lectin staining is also seen in the center of the ICH cavity, most likely due to blood brain barrier breakdown at this time point leading to nonspecific lectin staining of myeloid cells. 10x. White box indicates the inset shown in panel E. **D)** At 14 days, amoeboid, CX3CR1^+^ cells can still be seen in the perihematomal region, however more CX3CR1^+^ cells have a ramified morphology. No Ly6B.2^+^ (Ly6C^hi^ monocytes or neutrophils) cells are found. Overall blood-brain barrier disruption is improved leading to less lectin staining in the cavity, although there remain some lectin+ cells (CX3CR1^+^ monocytes and CX3CR1^−^ microglia). 10x. White box indicates inset shown in panel F. **E)** 20x image of panel C – perihematomal region 7 days after ICH **F)** 20x image of panel D – perihematomal region 14 days after ICH. Blue – Dapi, GFP – CX3CR1, Red – Tomato lectin, Pink – Ly6B.2, n = 5.

As shown in Figure 2, the staining protocol was confirmed to differentiate between lectin^+^ vasculature, CX3CR1^+^ peripherally-derived monocytes, and Ly6B.2^+^ inflammatory Ly6C^hi^ monocytes and neutrophils (n = 5). Amoeboid, CD11b^+^, CX3CR1^+^ peripherally-derived monocytes were in the tissue in the perihematomal region 3–14 days after ICH. While Ly6B.2^+^ cells (Ly6C^hi^ monocytes or neutrophils) were seen at day 3 (Figure 2B), the CX3CR1^+^ cells did not co-stain for Ly6B.2 on days 3, 7 or 14. This confirmed the CX3CR1^+^ cells visualized in the parenchyma were the Ly6C^lo^ population, not the Ly6C^hi^ population expressing low levels of CX3CR1 or neutrophils. Interestingly, the CX3CR1^+^ cells were not seen inside the vasculature but in the brain parenchyma. At 7 days, the majority of the amoeboid, CX3CR1^+^ monocytes were found surrounding the perimeter of the remaining hematoma, but not within it, as well as along the needle track (Figure 2C, E). At 14 days, amoeboid CX3CR1^+^ monocytes remained in the perihematomal region accompanied by an increase in ramified CX3CR1^+^ monocytes. These ramified and amoeboid CX3CR1^+^ monocytes were found throughout the perihematomal region, not just surrounding residual hematoma (Figure 2D, F). No difference in the number of CX3CR1^+^ monocytes was observed in the perihematomal region between 7 and 14 days.

### CX3CR1 signaling does not affect monocyte recruitment to the ipsilateral hemisphere after ICH

**Figure 3:**
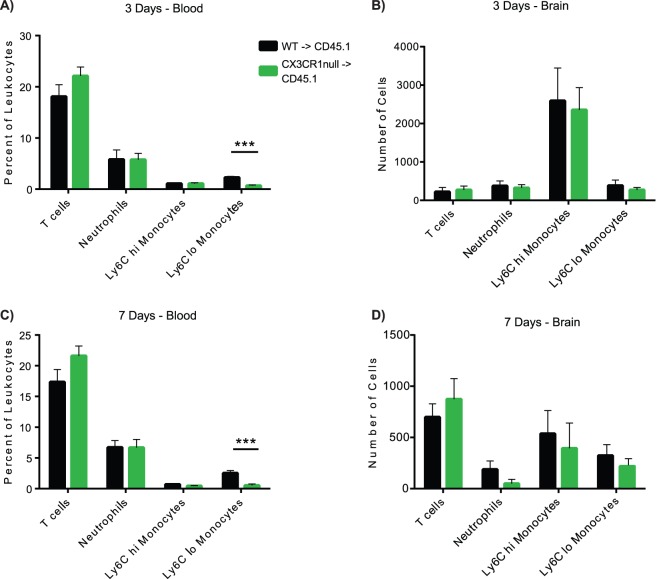
CX3CR1-deficiency on Ly6C^lo^ monocytes does not affect leukocyte recruitment to the ipsilateral hemisphere. **A)** Percent of leukocytes in the blood 3 days after ICH. CX3CR1 BM chimeras have a reduction in percentage of Ly6C^lo^ CX3CR1^+^ monocytes. **B)** At 3 days after ICH, WT BM chimeras and CX3CR1-null BM chimeras have equal numbers of T cells, Ly6C^hi^ monocytes, Ly6C^lo^ CX3CR1^+^ monocytes, and neutrophils in the ipsilateral hemisphere. The Ly6C^hi^ monocytes constitute the most numerous cell population recruited to the brain at 3 days in both genotypes. T cells, neutrophils, and Ly6C^lo^ CX3CR1^+^ monocytes are recruited in roughly equal numbers. n = 10 **C)** Percent of leukocytes in the blood 7 days after ICH. Similar to 3 days, the CX3CR1 BM chimeras have a smaller percentage Ly6C^lo^ CX3CR1^+^ monocytes in the blood. **D)** At 7 days, the numbers of T cells have increased, Ly6C^hi^ monocytes and neutrophils decreased, with no change in the numbers of Ly6C^lo^ CX3CR1^+^ monocytes. Like at 3 days, peripheral leukocyte populations do not differ between WT BM chimeras and CX3CR1-null chimeras. Bars indicate mean ± s.e.m. n = 9.

The peak of leukocyte recruitment is 72 hours after ICH [24]–[26], although some leukocyte subsets increase at later time points. To identify whether deficiency of CX3CR1 signaling on the peripheral leukocytes affects cellular recruitment of populations into the brain at the peak of inflammation, we first performed flow cytometry at 3 days after ICH. In the blood at 72 hours after ICH, there were no differences in the percent of T cells, neutrophils, or Ly6C^hi^ monocytes were seen between genotypes (n = 10) (Figure 3). The CX3CR1-null BM chimeras had a significant reduction in the percentage of the Ly6C^lo^, CX3CR1^+^ monocytes in the blood at 72 hours, which has been previously reported [27]. In the brains, no differences were seen in T cell, neutrophil, Ly6C^hi^ monocyte and Ly6C^lo^ monocyte recruitment between WT BM chimeras and CX3CR1-null BM chimeras, suggesting CX3CR1 deficiency on peripheral blood leukocytes does not affect cell recruitment after ICH.

The Ly6C^lo^, CX3CR1^+^ monocyte population infiltrates injured tissue after the Ly6C^hi^. CCR2^+^ monocytes, peaking at 7 days in models of spinal cord injury [10] and myocardial infarction [12]. We predicted that the recruitment of the Ly6C^lo^, CX3CR1^+^ monocytes would increase after the peak of inflammation, therefore, we examined leukocyte populations in the ipsilateral hemisphere at day 7. In the blood, we again observed no differences in percentages of T cells, neutrophils, and Ly6C^hi^ monocytes, with a similar reduction in the percentage of the Ly6C^lo^, CX3CR1^+^ monocytes (n = 9). Between days 3 and 7, both genotypes showed a decrease in neutrophil and Ly6C^hi^ monocyte numbers and an increase in T cells in the ipsilateral hemisphere. However, the numbers of Ly6C^lo^, CX3CR1^+^ monocytes in the ipsilateral hemisphere did not increase between 3 and 7 days. Interestingly, we did not observe a difference in the numbers of Ly6C^lo^, CX3CR1^+^ monocytes between the WT BM chimeras and the CX3CR1-null BM chimeras, despite the lack of CX3CR1 signaling. This suggests that CX3CR1 is not the chemokine receptor involved in their recruitment into the brain.

In order to determine whether CX3CR1-deficiency on monocytes affects cytokine production and cell surface marker expression 14 days after ICH, we then performed intracellular cytokine stain (ICS). By ICS, we could not detect either the Ly6C^lo^ or Ly6C^hi^ monocyte populations 14 days after ICH. We did not observe microglial TNF-α production 14 days after ICH in either genotype. We then measured phagocytic cell surface markers SIRPα and CD36. We did not observe differences in either SIRPα (WT: 882.4±49.33, CX3CR1-null: 860.0±99.37 a.u., p = 0.66, n = 5) or CD36 (WT: 1183±112.5, CX3CR1-null: 1496±411.6 a.u, p = 0.14, n = 5).

### CX3CR1 signaling on monocytes does not affect pro-inflammatory cytokine concentrations in the brain after ICH

CX3CR1 has been shown to regulate microglial neurotoxicity [6]. CX3CR1-null mice have worse functional outcomes after models of Parkinson’s disease, amyloid lateral sclerosis, and LPS-induced neuroinflammation [6]. In order to identify whether CX3CR1-deficiency on monocytes affects the inflammatory environment, we performed TNFα and IL-6 ELISA assays on ipsilateral brain homogenate in the WT and CX3CR1-null chimeras taken 7 days after ICH. We did not find a difference in concentrations of IL-6 in the ipsilateral hemisphere between genotypes (WT: 44.29±9.88, CX3CR1-null 57.03±19.78 pg/mL, n = 8 per group, p = 0.13). TNF was not detected in either genotype.

### CX3CR1 signaling on monocytes does not affect functional recovery after ICH

**Figure 4:**
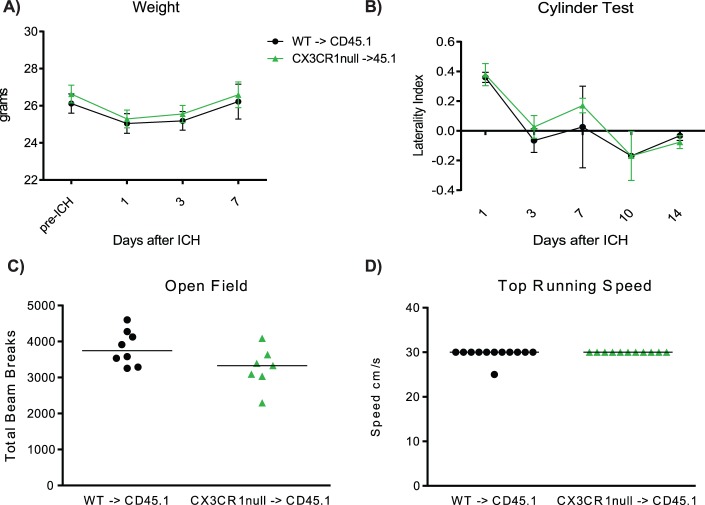
CX3CR1 deficiency on monocytes does not affect functional outcomes days 1–14 after ICH. **A)** WT BM chimeras and CX3CR1-null BM chimeras have similar weight loss and recovery. Means graphed with s.e.m.,n = 8–21, p>0.05 at all time points **B)** WT BM chimeras and CX3CR1-null BM chimeras have equal left forelimb weakness 24 hours after ICH and recover equally over the first 14 days by cylinder test. Means graphed with s.e.m., n = 8, p>0.05 at all time points **C)** Open field test shows CX3CR1 deficiency on monocytes does not impact spontaneous locomotor activity at 7 or 14 days after ICH. Line represents mean number of total beam breaks. n = 7–8, p>0.05 at each time point. **D)** CX3CR1-null BM chimeras perform equally well on the forced run test 7 and 14 days after ICH. Line represents median speed. n = 8–12, p>0.05 at each time point.

At baseline, there were no differences in behavior on cylinder test, open field, or top running speed between naïve, non-chimeric WT mice and CX3CR1-null mice, suggesting that the genetic background of the mice does not contribute to behavioral phenotype in the absence of injury (n = 3–9) (Figure S2). Twenty-four hours after ICH, both cohorts of chimeras had similar weight loss (WT 4.10±0.58% vs. CX3CR1-null 5.03±0.66%, n = 8–21, p = 0.30) (Figure 4). Both genotypes regained their original body weight by day 7. After ICH, the WT BM chimeras and CX3CR1-null BM chimeras demonstrated a similar acute right forelimb preference and complete improvement on days 3–14 by cylinder test (n = 8, p>0.05 at all time points). No differences in spontaneous locomotor activity or top running speed were observed between the WT BM chimeras and the CX3CR1-null BM chimeras on either 7 or 14 days (n = 8–12). Based on the similar neurobehavioral deficits during initial injury and recovery, we were able to identify no role for monocyte CX3CR1 signaling on functional outcomes after ICH.

## Discussion

The two murine monocyte subsets may be recruited to the brain after injury with different kinetics, and until now it was unclear whether Ly6C^lo^, CX3CR1^+^ monocytes would be found in the brain after ICH. Monocytes have been shown to enter the ipsilateral hemisphere as early as 12 hours after ICH [4]. Recently it has been shown that the Ly6C^hi^, CCR2^+^ monocytes contribute to acute injury after ICH [5]. The Ly6C^lo^, CX3CR1^+^ monocyte subset has been associated with wound repair and better functional outcome in models of spinal cord injury, myocardial infarction, and excitotoxic brain injury [10]–[13]. This contrasts with models of peritonitis and Listeria infection, in which the majority of the Ly6C^lo^, CX3CR1^+^ monocytes remain in the blood and do not enter inflamed tissues [27]. It has also been reported that the Ly6C^lo^ CX3CR1^+^ monocytes do not enter the brain after experimental autoimmune encephalomyelitis and that tissue remodeling is regulated by microglia [28]. We found that CX3CR1^+^, Ly6B.2^−^, peripherally-derived monocytes were in the perihematomal tissue, not in the vasculature, from 3–14 days after ICH (Table 1). Interestingly, at 7 days, the CX3CR1^+^ monocytes were found surrounding the perimeter of the residual hematoma rather than within it, while the Ly6B.2^+^ cells were found within the hematoma. This may suggest that the Ly6B.2^+^ cells, rather than CX3CR1^+^ monocytes, are involved in hematoma clearance. At 14 days, CX3CR1^+^ monocytes were found throughout the perihematomal region, possibly indicating their role in wound repair once the hematoma has been cleared. Furthermore, by 14 days, some CX3CR1^+^ monocytes have obtained a ramified, microglia-like morphology, an observation previously described of blood-derived Ly6C^hi^ CCR2^+^ monocytes after West Nile Virus infection [29].

**Table 1:** Experiments performed on WT and CX3CR1 BM chimeras after ICH.

Method	Days afterICH	Experiment	Results
Behavioral testing	1–14	Cylinder test, open field,and forced run test	No differences found in any behavioral test
Flow cytometry	3, 7	Quantification of peripheralleukocytes in the ipsilateralhemisphere	No differences found in recruitment of T cells, neutrophils, Ly6C^hi^ monocytes, or Ly6C^lo^ CX3CR1^+^ monocytes
ELISA	7	IL-6 and TNF on tissuehomogenate from ipsilateralhemisphere	No differences found in concentrations of IL-6. TNF was not detected
Immunohistochemistry	7, 14	Stain for blood vessels,Ly6B.2, CX3CR1, and Dapi	Amoeboid CX3CR1^+^ monocytes were observed at 7 days after ICH. Ramified CX3CR1^+^ cells were observed at 14 days.
Intracellular cytokine staining	14	Stain microglial for phagocyticmarkers CD36 and SIRPα andTNF-α	No differences observed between genotypes

The lack of CX3CR1 signaling on leukocytes did not affect peripheral leukocyte recruitment or functional recovery after ICH. We found that peripheral leukocytes were recruited into the ipsilateral hemisphere 3 and 7 days after ICH equally in chimeras with WT or CX3CR1-deficiency in the hematopoietic system. The peak of Ly6C^lo^ CX3CR1^+^ monocyte infiltration in other disease models is 7 days, when levels of CX3CL1 in injured tissue is elevated, suggesting the Ly6C^lo^ CX3CR1^+^ monocytes move along a CX3CL1 gradient [10], [12]. However, we found that after ICH, the population of Ly6C^lo^ CX3CR1^+^ monocytes in the ipsilateral hemisphere remains unchanged from 3 to 7 days. Furthermore, we found that despite the decrease in percentage of Ly6C^lo^ CX3CR1^+^ monocytes in the blood in the CX3CR1 BM chimeras, there was no difference in their recruitment to the ipsilateral hemisphere compared to WT on either day 3 or 7, demonstrating the signal for their recruitment remained intact. The CX3CR1-null BM chimeras had equal initial neurological deficit as WT BM chimeras and recovered equally by all four outcome tests performed after ICH. Rather, the point and variability estimates for each of the functional outcome tests at each time point are so similar between the genotypes that it is unlikely any difference that might exist would have any meaningful impact. Therefore we conclude there is no role for the chemokine receptor in functional outcomes after ICH. We used the whole blood injection model in this work, and a limitation of this model is that the initial neurological deficit after ICH is not as severe as in the collagenase model. However, the introduction of bacterial collagenase into the brain parenchyma may result in inflammation due to the presence of a foreign antigen in the brain [20], which is not a concern with the blood injection model.

Previous reports suggest that the Ly6C^lo^ CX3CR1^+^ monocytes may be recruited sequentially after the Ly6C^hi^ CCR2^+^ [10], [12] or that they may differentiate from the Ly6C^hi^ population *in situ* [30], [31]. Our data suggests that if the Ly6C^lo^ CX3CR1^+^ monocytes enter the brain directly after ICH, it is in a CX3CR1-independent manner. This is consistent with the work in spinal cord injury, in which the Ly6C^lo^, CX3CR1^+^ monocytes traffic to the cord via the brain choroid plexus, peaking at day 7. Interrupting their migration via inhibition of either CD73 or the VCAM-1-VLA-4 interaction reduced Ly6C^lo^ monocyte recruitment, decreased IL-10 production, and worsened outcomes from a few days to several weeks after injury [10]. It may also be possible that the Ly6C^lo^ monocytes are recruited to the ipsilateral hemisphere via other chemokines, including CXCL10 [32] or CCL3 [33], as has been shown in other disease models. Alternatively, the Ly6C^lo^, CX3CR1^+^ macrophages in the perihematomal brain could have differentiated from a Ly6C^hi^ CCR2^+^ population that has downregulated Ly6C and upregulated CX3CR1. Definitively determining the source of the CX3CR1^+^ cells and their interactions with other leukocyte populations is beyond the scope of this work.

Recently, it has been shown that the Ly6C^lo^ CX3CR1^+^ monocytes did not impact functional recovery or severity of injury in a murine model of ischemic stroke [34]. That work utilized Nr4a1^−/−^ chimeras, which lack the transcription factor required for Ly6C^lo^ monocyte development. We took an alternative approach, and focused on the principle chemokine receptor of the Ly6C^lo^ monocytes, and found similar results after ICH. While we cannot be certain of the role of Ly6C^lo^ monocytes after ICH, our work demonstrates that CX3CR1-null mice are not an adequate tool to investigate their function in this disease. Identifying the origin of the CX3CR1^+^ cells or using other methods to enhance or inhibit their entry into the brain parenchyma may help elucidate a potential role in injury or recovery after ICH, and may be a focus for future work.

Taken together, our results indicate that Ly6C^lo^, CX3CR1^+^ macrophages are present in the perihematomal region after ICH but (1) their recruitment is independent of CX3CR1 and (2) that CX3CR1 signaling does not affect functional recovery after ICH. We conclude that CX3CR1 is not a target to improve outcomes after ICH.

## Supporting Information

Figure S1 **Method to calculate percent chimerism from blood samples.** Leukocytes are gated on live and singlets prior to being located by forward and side scatter. Samples are then gated CD45.1 by CD45.2. Chimerism is calculated by dividing quadrant 1 (CD45.2^+^, CD45.1^−^) by the sum of quadrant 1 (CD45.2^+^, CD45.1^−^) and quadrant 3 (CD45.2^−^, CD45.1^+^). Percent chimerism = (Q1/(Q1+Q3)). Quadrant 1 and quadrant 3 are outlined in red boxes.
(EPS)

Figure S2 **CX3CR1 deficiency does not affect functional outcomes at baseline.** **A)** WT mice and CX3CR1 mice have similar initial weights. Means graphed with s.d. n = 8 **B)** WT mice and CX3CR1 mice do not have a forelimb preference at baseline. Means graphed with s.e.m., n = 8–9 **C)** Open field test shows CX3CR1 deficiency on monocytes does not impact spontaneous locomotor activity at baseline. n = 3–9 **D)** CX3CR1-null BM chimeras perform equally well on the forced run test at baseline. 7 and 14 days after ICH. Line represents median speed. n = 9.
(EPS)
